# Does treatment with antidepressants, antipsychotics, or benzodiazepines hamper allergy skin testing?

**DOI:** 10.1002/clt2.12060

**Published:** 2021-09-06

**Authors:** Henrik Fomsgaard Kjaer, Charlotte Gotthard Mortz, Carsten Bindslev‐Jensen

**Affiliations:** ^1^ Department of Dermatology and Allergy Centre Odense Research Center for Anaphylaxis (ORCA) Odense University Hospital Odense Denmark

**Keywords:** allergy diagnosis, antidepressants, antihistaminic, antipsychotics, benzodiazepines, H_1_‐receptor, mirtazapine, quetiapine, skin prick test, suppression

## Abstract

**Background:**

Treatment with commonly used drugs such as antidepressants (ADs), antipsychotics (APs), and benzodiazepines (BDs) may hamper the use of allergy skin testing due to possible antihistaminic effects.

**Objective:**

To examine the antihistaminic effect of AD, AP, and BD as measured by the ability of these drugs to suppress the normal wheal reaction caused by skin prick test (SPT).

**Methods:**

Skin prick test was performed in patients receiving treatment with AD, AP, and/or BD. Double SPT was performed with histamine solutions of 10, 30, and 100 mg/ml and mean wheal diameter calculated.

**Results:**

A total of 313 patients were included. 236 (75%) patients were treated with one of the examined drugs and 77 (25%) patients with more than one of these drugs. Drugs most frequently used was sertraline (*n* = 65), citalopram (*n* = 63), mirtazapine (*n* = 36), venlafaxine (*n* = 33), and quetiapine (*n* = 32). Treatment with mirtazapine and/or quetiapine was associated with negative SPTs in 30/36 (83%) and 22/32 (69%), and the antihistaminic effect of these drugs was dose‐dependent. For patients treated with selective serotonin reuptake inhibitors (SSRIs), tricyclic antidepressants (TCAs), serotonin norepinephrine reuptake inhibitors (SNRIs), or BD alone, almost all SPTs were positive (94%, 95%, 100%, and 100%, respectively). Negative SPTs in patients treated with SSRI, TCA, SNRI, or BD and ≥1 other of the examined drugs were associated with simultaneous treatment with mirtazapine or quetiapine in 39/44 (89%) patients.

**Conclusion:**

Skin testing has little meaning in patients treated with mirtazapine or quetiapine. Treatment with SSRI, SNRI, and BD does not seem to affect the results of SPTs, whereas skin tests in patients treated with TCA should be interpreted with caution.

## 1 INTRODUCTION

Treatment with antidepressants (ADs), antipsychotics (APs), and/or benzodiazepines (BDs) is very common, and many patients referred for allergy evaluation are in ongoing treatment with these drugs. In Denmark with a total population of 5.8 million people (4.5 million adults), the number of patients treated with AD, AP, or BD in 2019 was 420,000, 130,000 and 258,000.^1^ Since up to 40% of the general population suffer from allergic diseases, the overlap between treatment with these drugs and need for allergy evaluation is large.^2^ Ongoing treatment with AD, AP, or BD can complicate the diagnostic work‐up for allergy due to the potential antihistaminic effect of these drugs. Skin prick tests (SPTs) are based on interpretation of wheal and flare reactions caused by the release of histamine in the skin to allergens the patient do not tolerate.^3^ The test can however not be used if treatment with ongoing drugs suppresses this histamine release. A very important part of evaluation for food‐ and drug allergy is allergen challenges. These challenges should however not be performed if the patient receives drugs with antihistaminic effect since this hampers interpretation of the challenge outcome.

Suppression of skin test reactivity has been studied for different drugs such as oral antihistamines,^4^, ^5^ oral steroids,^6^, ^7^, ^8^ topical steroids, and calcineurin inhibitors^9^, ^10^ as well as other drugs.^3^ The very few published studies on AD, AP, and BD are small, rather old and retrospective.^11^, ^12^, ^13^ As such, the knowledge on the degree of the specific antihistaminic effect of different ADs, APs, and BDs used nowadays is sparse.

The aim of this study was to examine the antihistaminic effect of different ADs, APs, and BDs as measured by the ability of the drug to suppress the normal wheal and flare reaction caused by SPT with histamine in patients using these drugs.

## 2 METHODS

Adult patients (age ≥ 18 years) referred to the Allergy Centre, Odense University Hospital, Denmark for allergy evaluation were asked to participate in this observational, cross‐sectional study provided they received ongoing treatment with AD, AP, or BD. The inclusion period was from November 2017 to April 2019. All drugs (including doses) received by the patient were recorded by the doctor, and it was verbally assured that the patient was actually compliant in taking these drugs. All patients referred to our Allergy Centre receive a letter before their visit asking them to discontinue any use of oral antihistamines at least 72 h before their visit. At the visit, it was reassured that the patient had followed these instructions on discontinuation of oral antihistamines—and that the patient did not use oral steroids—or potent topical steroids on the forearms. An SPT with a histamine solution of 10, 30, and 100 mg/ml (twice for each concentration) and a negative control with saline was performed on the forearm of the patient. The size of the resulting wheals was recorded after 15 min, and the wheal size was measured on the longest and the midpoint orthogonal diameter; the numbers were added and divided by two to calculate the mean wheal diameter.^14^ Finally, the mean from the two tests on each concentration was recorded. The antihistaminic effect of specific drugs was determined from these readings. In addition to patients fulfilling the above‐specified inclusion criteria, 10 healthy participants not using any drugs (nurses/doctors working in the department) were also skin prick tested. Median wheal size and quartiles (Q1: first quartile, Q3: third quartile) as well as significance testing between the control group and different drug groups were performed in STATA/SE 16.0 (Stata Corporation), the latter analyses using the two sample Wilcoxon rank‐sum (Mann–Whitney *U*) test.

### 2.1 Ethics

The study was approved by the Regional Committees on Health Research Ethics for Southern Denmark (project ID S‐20160141) and the Danish Data Protection Agency (no. 18/54980). Oral and written informed consent was obtained from all patients.

## 3 RESULTS

A total of 313 patients receiving treatment with AD, AP, and/or BD were included. All patients were skin prick tested. Table 1 (drugs with ≥5 patients treated) and Table 2 (drugs with <5 patients treated) list specific drugs and number of treated patients. Of the 313 patients, 236 (75%) patients were treated with one of the examined drugs and 77 (25%) patients with more than one of these drugs. Of the latter, 60 patients were treated with two drugs, 16 with three drugs, and 1 patient with four drugs. Median age of participants was 55 years with an age span of 20–90 years.

**TABLE 1:** Skin prick test results (histamine solution of 10 mg/ml) for drugs with ≥5 patients treated

	*n*	No wheal	0 < SPT < 3 mm	3 ≤ SPT < 5 mm	SPT ≥ 5 mm	SPT ≥ 3 mm
Healthy controls	10	0	0	1 (10%)	9 (90%)	10 (100%)
SSRI						
Sertraline alone	60	2 (3%)	2 (3%)	10 (17%)	46 (77%)	56 (93%)
Sertraline combination	5	2 (40%)	2 (40%)	0	1 (20%)	1 (20%)
Citalopram alone	47	0	3 (6%)	7 (15%)	37 (79%)	44 (94%)
Citalopram combination	16	9 (56%)	0	0	7 (44%)	7 (44%)
Escitalopram alone	5	0	0	1 (20%)	4 (80%)	5 (100%)
Escitalopram combination	4	2 (50%)	0	0	2 (50%)	2 (50%)
Paroxetine alone	7	0	0	0	7 (100%)	7 (100%)
Paroxetine combination	1	1 (100%)	0	0	0	0
Fluoxetine alone	4	0	0	0	4 (100%)	4 (100%)
Fluoxetine combination	3	2 (67%)	0	1 (33%)	0	1 (33%)
TCA						
Amitriptyline alone	10	1 (10%)	0	4 (40%)	5 (50%)	9 (90%)
Amitriptyline combination	10	1 (10%)	0	2 (20%)	7 (70%)	9 (90%)
Nortriptyline alone	7	0	0	1 (14%)	6 (86%)	7 (100%)
Nortriptyline combination	3	0	1 (33%)	0	2 (67%)	2 (67%)
Imipramine alone	3	0	0	0	3 (100%)	3 (100%)
Imipramine combination	3	0	0	1 (33%)	2 (67%)	3 (100%)
SNRI						
Venlafaxine alone	21	0	0	3 (14%)	18 (86%)	21 (100%)
Venlafaxine combination	12	4 (33%)	0	2 (17%)	6 (50%)	8 (67%)
Duloxetine alone	12	0	0	3 (25%)	9 (75%)	12 (100%)
Duloxetine combination	13	3 (29%)	1 (7%)	3 (21%)	6 (43%)	9 (69%)
NaSSA						
Mirtazapine alone	15	13 (87%)	0	0	2 (13%)	2 (13%)
Mirtazapine combination	21	15 (71%)	2 (10%)	2 (10%)	2 (10%)	4 (19%)
Benzodiazepines						
Oxazepam alone	5	0	0	1 (20%)	4 (80%)	5 (100%)
Oxazepam combination	12	6 (50%)	1 (8%)	0	5 (42%)	5 (42%)
Zopiclone alone	8	0	0	0	8 (100%)	8 (100%)
Zopiclone combination	10	4 (40%)	0	0	6 (60%)	6 (60%)
Zolpidem alone	4	0	0	0	4 (100%)	4 (100%)
Zolpidem combination	10	5 (50%)	0	1 (10%)	4 (40%)	5 (50%)
Antipsychotics						
Quetiapine alone	8	1 (13%)	2 (25%)	3 (38%)	2 (25%)	5 (63%)
Quetiapine combination	24	14 (58%)	5 (21%)	3 (13%)	2 (8%)	5 (21%)
Chlorprothixen alone	4	0	0	1 (25%)	3 (75%)	4 (100%)
Chlorprothixen combination	6	2 (33%)	1 (17%)	1 (17%)	2 (33%)	3 (50%)
Aripiprazol alone	0	‐	‐	‐	‐	‐
Aripiprazol combination	5	0	2 (40%)	2 (40%)	1 (20%)	3 (60%)
Risperidon alone	1	0	0	0	1 (100%)	1 (100%)
Risperidon combination	4	0	0	0	4 (100%)	4 (100%)

Abbreviations: NaSSA, noradrenergic and specific serotonergic antidepressants; SNRIs, serotonin norepinephrine reuptake inhibitors; SPT, skin prick test; SSRIs, selective serotonin reuptake inhibitors; TCAs, tricyclic antidepressants.

**TABLE 2:** Skin prick test results (histamine solution of 10 mg/ml) for drugs with <5 patients treated

	*n*	No wheal	0 < SPT < 3 mm	3 ≤ SPT < 5 mm	SPT ≥ 5 mm	SPT ≥ 3 mm
Antipsychotics						
Clozapine alone	2	2 (100%)	0	0	0	0
Clozapine combination	0	‐	‐	‐	‐	‐
Flupentixol alone	0	‐	‐	‐	‐	‐
Flupentixol combination	1	0	0	0	1 (100%)	1 (100%)
Levomepromazine alone	1	0	0	0	1 (100%)	1 (100%)
Levomepromazine comb.	0	‐	‐	‐	‐	‐
Olanzapine alone	1	1 (100%)	0	0	0	0
Olanzapine combination	0	‐	‐	‐	‐	‐
Ziprasidone alone	1	0	0	1 (100%)	0	1 (100%)
Ziprasidone combination	0	‐	‐	‐	‐	‐
Zuclopenthixol alone	0	‐	‐	‐	‐	‐
Zuclopenthixol combination	1	1 (100%)	0	0	0	0
Serotonin modulators						
Vortioxetine alone	3	0	0	0	3 (100%)	3 (100%)
Vortioxetine combination	0	‐	‐	‐	‐	‐
NaSSA						
Mianserine alone	0	‐	‐	‐	‐	‐
Mianserine combination	2	1 (50%)	0	0	1 (50%)	1 (50%)
Benzodiazepines						
Alprazolam alone	3	0	0	0	3 (100%)	3 (100%)
Alprazolam combination	0	‐	‐	‐	‐	‐
Chlordiazepoxide alone	1	0	0	0	1 (100%)	1 (100%)
Chlordiazepoxide combination	0	‐	‐	‐	‐	‐
Diazepam alone	2	0	0	1 (50%)	1 (50%)	2 (100%)
Diazepam combination	1	0	0	1 (100%)	0	1 (100%)
Lorazepam alone	0	‐	‐	‐	‐	‐
Lorazepam combination	2	1 (50%)	0	0	1 (50%)	1 (50%)
Lormetazepam alone	0	‐	‐	‐	‐	‐
Lormetazepam combination	1	0	0	1 (100%)	0	1 (100%)
Triazolam alone	1	0	0	1 (100%)	0	1 (100%)
Triazolam combination	0	‐	‐	‐	‐	‐

Abbreviations: NaSSA, noradrenergic and specific serotonergic antidepressants; SPT, skin prick test.

To begin with, we looked at the AD, AP, and BD most frequently used (≥5 patients treated). SPTs were evaluated separately for patients receiving only one of these drugs (“alone”) as opposed to patients receiving more than one of these drugs (“combination”). The result of the SPT (wheal size) was categorized into “no wheal,” 0 < SPT < 3 mm, 3 ≤ SPT < 5 mm, and SPT ≥ 5 mm. An SPT < 3 mm is hereafter referred to a “negative.” The two latter categories were also combined (see Tables 1 and 2) since an SPT ≥ 3 mm is defined as a positive SPT provided the saline control is negative. All saline controls were negative.

The most frequently taken drugs were sertraline (*n* = 65), citalopram (*n* = 63), mirtazapine (*n* = 36), venlafaxine (*n* = 33), quetiapine (*n* = 32), duloxetine (*n* = 27), amitriptyline (*n* = 20), zopiclone (*n* = 18), and oxazepam (*n* = 17). Treatment with most of these substances alone resulted in positive SPTs (Table 1). SPT was positive in 116/123 (94%) treated with selective serotonin reuptake inhibitors (SSRIs) alone, 19/20 (95%) (tricyclic antidepressants [TCAs] alone), 33/33 (100%) (serotonin norepinephrine reuptake inhibitors [SNRIs] alone), and 17/17 (100%) (BD alone). Ongoing treatment with mirtazapine and quetiapine did however suppress the skin test response. Patients treated with mirtazapine alone had a positive SPT in 2/15 (13%) cases, while the number for quetiapine was 5/8 (63%).

In the drug groups where monotherapy very rarely resulted in negative SPTs, combined therapy did so to a much higher degree. We therefore looked into specific combinations of the drugs and especially evaluated if simultaneous treatment with mirtazapine or quetiapine could explain negative SPTs in the groups treated with SSRI, SNRI, TCA, and BD. In the SSRI combination therapy group, 16/18 (89%) patients with a negative SPT were also treated with either mirtazapine or quetiapine. Negative SPTs in patients with combination therapy in the groups of TCA, SNRI, and BD could be explained by the simultaneous use of mirtazapine or quetiapine in 2/2 (100%), 7/8 (89%), and 14/16 (88%), respectively.

Treatment with mirtazapine and quetiapine was associated with almost all negative SPTs in the study, but a few patients did have a positive SPT despite treatment with mirtazapine or quetiapine. Of the 36 patients treated with mirtazapine, six (17%) patients had a positive SPT; of the 32 patients treated with quetiapine, 10 (31%) patients had a positive SPT (Table 1). Suppression of the SPT by mirtazapine was clearly dose‐dependent; patients on a daily dose of 7.5 mg had a positive SPT in 2/4 (50%) cases, whereas patients on a daily dose of 15, 30, or 45 mg had positive SPTs in 3/16 (19%), 1/11 (9%), and 0/5 (0%). The exact same was seen for quetiapine, patients treated with a daily dose of 25 mg had a positive SPT in 7/10 (70%) cases, whereas patients on a daily dose of 50, 100, 150, 200, 500, 600, or 750 mg had positive SPTs in 2/7 (29%), 1/4 (25%), 0/2 (0%), 0/4 (0%), 0/3 (0%), 0/1 (0%), and 0/1 (0%), respectively.

Dose dependency of all other drugs rarely associated with negative SPTs was also evaluated, but no association was found neither between dose and frequency of negative SPTs nor between dose and wheal size (data not shown).

In addition to standardized SPT with a histamine concentration of 10 mg/ml, SPT was also performed with histamine concentrations of 30 and 100 mg/ml. This was done to evaluate whether the suppressed SPTs with the standardized 10 mg/ml concentration would be positive with a higher histamine concentration. Overall, in the 313 SPTs, the median wheal diameter (with first and third quartiles) for 10, 30, and 100 mg/ml was 5.5 [4.0:6.5], 6.3 [4.5:7.8], and 7.3 [5.3:8.8], respectively.

Of the 313 patients, 14 (4%) patients had negative SPTs, which could not be explained by treatment with mirtazapine or quetiapine. Of those, four were in monotherapy with sertraline (2/4 positive in the histamine concentration of 30 mg/ml), three in monotherapy with citalopram (2/3 positive in the histamine concentration of 30 mg/ml), one in monotherapy with amitriptyline (positive in the histamine concentration of 100 mg/ml), two in monotherapy with clozapine (one positive in 30 mg/ml), and one in monotherapy with olanzapine (negative also in 30 and 100 mg/ml). The last 3/14 had combined escitalopram + zolpidem, citalopram + zopiclone and mianserine + venlafaxine + lorazepam, and these three patients were negative also in 30 and 100 mg/ml. As such, apart from treatment including mirtazapine or quetiapine, we did not find significant evidence that specific combinations of other drugs suppressed the histamine response in any pattern.

Mirtazapine was also a highly significant suppressor of the histamine response when evaluating median wheal size for monotherapy compared to controls (*p* = 0.0004) (Table 3). Median wheal size for quetiapine (4.4 mm) and amitriptyline (4.9 mm) was also reduced compared to the control group, but the differences did just not reach a significance level of *p* < 0.05 (*p* = 0.06 and 0.07, respectively). Median wheal size for the drug groups of SSRI, SNRI, and BDs was not reduced compared to the control group, but for zopiclone it was actually increased. Median age differed somewhat between the groups, whereas the gender ratio was comparable.

**TABLE 3:** Age, sex, median wheal size (for histamine 10 mg/ml) and quartiles for drugs used in monotherapy (≥5 treated patients) and healthy controls (*p < 0.05, **p < 0.01, ***p < 0.001 for comparison with healthy controls)

	*n*	Age median + [Q1:Q3] (years)	Female, *n* (%)	Wheal size median + [Q1:Q3] (mm)
Healthy controls	10	44 [35:55]	8 (80)	5.5 [5.0:6.0]
SSRI				
Sertraline alone	60	43 [32:62]	53 (88)	5.8 [5.0:6.9]
Citalopram alone	47	57 [44:67]*	34 (72)	5.8 [5.0:6.8]
Escitalopram alone	5	54 [53:55]	5 (100)	6.5 [5.0:7.8]
Paroxetine alone	7	56 [49:66]	6 (86)	6.0 [5.8:7.5]
TCA				
Amitriptyline alone	10	65 [50:68]*	8 (80)	4.9 [3.8:5.3]
Nortriptyline alone	7	60 [48:79]*	6 (86)	5.3 [5.0:5.5]
SNRI				
Venlafaxine alone	21	44 [32:60]	18 (86)	6.0 [5.5:6.3]
Duloxetine alone	12	51 [42:62]	10 (83)	6.1 [4.9:7.5]
NaSSA				
Mirtazapine alone	15	58 [54:69]**	11 (73)	0.0 [0.0:0.0]***
Benzodiazepines				
Oxazepam alone	5	59 [58:62]*	3 (60)	6.8 [6.0:7.3]
Zopiclone alone	8	66 [53:78]*	5 (63)	7.1 [5.5:8.0]*
Antipsychotics				
Quetiapine alone	8	45 [40:53]	6 (75)	4.4 [1.9:5.4]

Abbreviations: NaSSA, noradrenergic and specific serotonergic antidepressants; SNRIs, serotonin norepinephrine reuptake inhibitors; SSRIs, selective serotonin reuptake inhibitors; TCAs, tricyclic antidepressants.

## 4 DISCUSSION

Although some limitations have to be taken into account, our study revealed quite clear‐cut results. Almost all negative SPTs could be explained by treatment with mirtazapine or quetiapine, the antihistaminic effect of these drugs being dose‐dependent. Mirtazapine was by far the most efficacious suppressor of the histamine response. Mirtazapine exhibits well‐known potent antagonism of H_1_ receptors. A single 15‐mg dose of mirtazapine to healthy volunteers has been found to result in over 80% occupancy of the H_1_ receptor.^15^ In the study by Shah et al., both mirtazapine and quetiapine were included in the group of atypical ADs (along with bupropion, eszopiclone, trazodone, and zolpidem) in which 92.6% of the patients had a positive SPT. However, only four patients were treated with mirtazapine and all of them had negative SPTs, whereas 8/11 (73%) patients treated with quetiapine had no skin test reactivity.^13^ In our study, 30/36 (83%) patients treated with mirtazapine and 22/32 (69%) treated with quetiapine had negative SPTs. Median wheal size for quetiapine monotherapy did however not differ significantly from controls (*p* = 0.06). One explanation for this might be that low‐dose quetiapine treatment was more frequent in the monotherapy group (median dose: 25 mg, Q1: 25 mg, and Q3: 50 mg), as opposed to the quetiapine combination therapy group (median dose: 100 mg, Q1: 50 mg, and Q3: 200 mg). Another explanation might be the rather small groups of both the Quetiapine monotherapy group and the control group as discussed further below. As mirtazapine, quetiapine is a well‐known H_1_ receptor antagonist.^16^ A Japanese study using positron emission tomography (PET) imaging studies with [11C]doxepin, a potent PET(positron emission tomography) ligand of the H_1_ receptor, demonstrated that even very small doses of quetiapine (and olanzapine) were able to block more than 60% of brain H_1_ receptors.^17^

In our study, the antihistaminic effect of both mirtazapine and quetiapine was clearly dose‐dependent. The vast majority of patients having a positive SPT, despite ongoing treatment with these drugs, received low‐dose treatment and their positive SPTs undoubtedly reflect incomplete occupancy of the H_1_ receptor. We considered alternative explanations for positive SPTs in patients receiving mirtazapine or quetiapine such as concomitant medication affecting the metabolism of these drugs in the liver (i.e., partial occupation of the CYP3A4 enzyme reducing the metabolism of mirtazapine/quetiapine) but no pattern was found.

A few older studies looked at the effect of different TCAs on the H_1_ receptor in animal models and described antagonism to various degrees, most prominently for doxepin.^18^ A human study examining the effect of a single dose of doxepin and desipramine demonstrated the partial suppression of the skin test response for 4 days after the administration of doxepin, whereas for desipramin a slight suppression for 2 days was seen.^11^ Newer data resulting from assays using human cloned receptors also reveal various H_1_ receptor antagonism for the different TCAs compared to mirtazapine and mianserine: Amitriptyline shows lower potency for the H_1_ receptor than mirtazapine, mianserine, and doxepin, but higher potency for the receptor than nortriptyline and imipramine.^19^ In our study, treatment with amitriptyline, nortriptyline, or imipramin was associated with a positive SPT in 33/36 cases; 2/3 of the negative SPTs being associated with treatment with mirtazapine or quetiapine. This is in contrast to the Shah study with a comparable number of patients on amitriptyline/nortriptyline with just over half of their patients having a positive histamine control. Although, almost all patients on TCA in our study had a positive SPT, the median wheal size for amitriptyline (4.9 mm) was smaller than the median wheal size of the control group. This difference did however not reach statistical significance (*p* = 0.07), but taken into account the limitation of small groups, the difference could possibly reflect a partial suppression of the histamine release.

The Shah study suggested that also patients treated with BD should have their BD temporarily discontinued before skin testing if clinically able, although 85.7% of only seven patients on “single medication” had a positive SPT. In their multivariate regression analysis, however, they reported an odds ratio of 5 (1.72–15.8) for a negative histamine control for patients receiving BD. They included the BDs, clonazepam, diazepam, lorazepam, and midazolam, in their study.^13^ In our study, all 24 participants on BD monotherapy (mainly oxazepam, zopiclone, and zolpidem) had a positive skin test response, moreover with mean wheal sizes larger than our control group and in conclusion therefore not suggestive of any suppression of the histamine response.

Since all participants tested did receive ongoing treatment with the specified drugs, we do not know what results the SPTs would have revealed for each individual if these drugs were discontinued. As such, in this study, patients do not serve as their own controls. This is of course a limitation when evaluating the direct influence of the medication in detail; on the other hand, discontinuation of these kind of drugs would be quite a challenge in a study setting, in some cases even unethical.

Given this, the comparison between wheal sizes in groups of monotherapy and controls might represent the best way of looking into the ability of the different drugs to suppress the histamine response. Skin test positivity (wheal ≥ 3 mm) alone is likely too crude an outcome measure since a wheal may be positive, but still suppressed from 5 to 3 mm. For this reason, we presented crude wheal size data in different categories (no wheal, 0 < wheal < 3 mm, 3 ≤ wheal < 5 mm and wheal ≥ 5 mm). However, in the comparison between median wheal sizes between monotherapy treatment and controls, the size of our control group probably represents the most important limitation to our study. A larger control group, maybe even age comparable to a higher extent with the drug monotherapy groups, would have made conclusions of the study stronger. Some drug groups were also rather small, but this merely reflected the tradition of prescription in the community. Finally, the fact that SPTs to some extent were read by different nurses might introduce some interobserver variability. Due to standardized quality control measures regarding SPT variability in our Allergy Center, we do however regard this variability as quite low. Age differed somewhat between monotherapy treatment groups and controls in comparison with median wheal size. Some studies have described reduced skin test reactivity of elderly patients.^20^ Age was significantly higher in monotherapy groups of mirtazapine, amitriptyline, and nortriptyline compared to controls and represents a possible confounder, but age was also significantly higher in monotherapy groups of citalopram, oxazepam, and zopiclone with higher median wheal values than the control group.

Although not specifically evaluated in this study, allergen challenges should not be performed if the patient receives ongoing antihistaminic drugs, since this hampers interpretation of the challenge result. Since discontinuation of the examined drugs was not part of our study, our data cannot be used to recommend for how many days mirtazapine or quetiapine should be discontinued before an interpretable SPT or allergen challenge could be performed. Such recommendations rely on the specific half‐life of different drugs. Normally patients on different kinds of antihistamines can discontinue their treatment for the recommended duration (often 3–5 days) allowing for SPT or challenges to be performed. Discontinuation of treatment with mirtazapine or quetiapine should however be done gradually over several weeks to avoid discontinuation symptoms. Abrupt discontinuation of ADs and AP should not be done—and if deemed necessary for allergy diagnostic work‐up with SPTs and/or allergen challenges, the discontinuation should be discussed with the prescribing provider of these drugs. Measurement of specific IgE (immoglobulin E) is not affected by treatment with antihistaminic drugs and can be used to guide advice and the need for further evaluation in the allergic patient receiving drugs where SPTs and allergen challenges are challenging due to the aforementioned reasons.

In conclusion, this study presents clinical data from a large cohort describing the association between treatment with different ADs, APs, and/or BDs and SPT results. Based on our results and the associated pharmacologic knowledge, we suggest that SPTs (and allergen challenges) have little meaning if the patient is treated with mirtazapine or quetiapine due to the antihistaminic effect of these drugs. SPT might be considered in patients treated with very low doses of mirtazapine and quetiapine, but results should be interpreted very carefully. SPTs in patients receiving TCA can, according to our results, be performed, but interpretation should be done with caution, since these drugs may well partially suppress the wheal response. For AP other than quetiapine and a number of older BD, the groups were too small to draw conclusions, but crude data have been presented. Our data suggest that treatment with SSRI, SNRI, and most of the BD used nowadays is unlikely to interfere with allergy skin testing (and/or allergen challenges)—meaning these procedures can be performed without discontinuation of these drugs.

## CONFLICT OF INTEREST

The authors declare that they have no conflict of interest.

## AUTHOR CONTRIBUTIONS

Henrik Fomsgaard Kjaer: Conceptualization; Lead, Data curation; Lead, Formal analysis; Lead, Investigation; Lead, Methodology; Equal, Project administration; Lead, Resources; Equal, Validation; Lead, Visualization; Lead, Writing‐original draft; Lead. Charlotte Gotthard Mortz: Conceptualization; Supporting, Data curation; Supporting, Formal analysis; Equal, Investigation; Supporting, Methodology; Equal, Project administration; Supporting, Resources; Supporting, Supervision; Equal, Validation; Supporting, Writing‐original draft; Supporting. Carsten Bindslev‐Jensen: Conceptualization; Lead, Data curation; Supporting, Formal analysis; Equal, Investigation; Equal, Methodology; Equal, Project administration; Supporting, Resources; Equal, Supervision; Equal, Validation; Supporting, Writing‐original draft; Supporting.
